# Application of an objective structured clinical examination to evaluate and monitor interns’ proficiency in hand hygiene and personal protective equipment use in the United States

**DOI:** 10.3352/jeehp.2019.16.31

**Published:** 2019-10-15

**Authors:** Ying Nagoshi, Lou Ann Cooper, Lynne Meyer, Kartik Cherabuddi, Julia Close, Jamie Dow, Merry Jennifer Markham, Carolyn Stalvey

**Affiliations:** 1 Division of General Internal Medicine, Department of Medicine, University of Florida, Gainesville, FL, USA; 2 College of Medicine Educational Affairs, University of Florida, Gainesville, FL, USA; 3 Graudate of Medical Education, College of Medicine, University of Florida, Gainesville, FL, USA; 4 Division of Infectious Diseases, Department of Medicine, University of Florida, Gainesville, FL, USA; 5 Division of Hematology and Oncology, Department of Medicine, University of Florida, Gainesville, FL, USA; 6 Department of Neurosurgery, University of Florida, Gainesville, FL, USA; Hallym University, Korea

**Keywords:** Cohort studies, Hand hygiene, Personal protective equipment, United States

## Abstract

**Purpose:**

This study was conducted to determine whether an objective structured clinical examination (OSCE) could be used to evaluate and monitor hand hygiene and personal protective equipment (PPE) proficiency among medical interns in the United States.

**Methods:**

Interns in July 2015 (N=123, cohort 1) with no experience of OSCE-based contact precaution evaluation and teaching were evaluated in early 2016 using an OSCE for hand hygiene and PPE proficiency. They performed poorly. Therefore, the new interns entering in July 2016 (N=151, cohort 2) were immediately tested at the same OSCE stations as cohort 1, and were provided with feedback and teaching. Cohort 2 was then retested at the OSCE station in early 2017. The Mann-Whitney U-test was used to compare the performance of cohort 1 and cohort 2 on checklist items. In cohort 2, performance differences between the beginning and end of the intern year were compared using the McNemar chi-square test for paired nominal data.

**Results:**

Checklist items were scored, summed, and reported as percent correct. In cohort 2, the mean percent correct was higher on the posttest than on the pretest (92% vs. 77%, P<0.0001), and the passing rate (100% correct) was also significantly higher on the posttest (55% vs. 16%). At the end of intern year, the mean percent correct was higher in cohort 2 than in cohort 1 (95% vs. 90%, P<0.0001), and 55% of cohort 2 passed (a perfect score) compared to 24% in cohort 1 (P<0.0001).

**Conclusion:**

An OSCE can be utilized to evaluate and monitor hand hygiene and PPE proficiency among interns in the United States.

## Introduction

### Background

Contact precautions have been instrumental in reducing the incidence of hospital-acquired *Clostridioides difficile* and infections with multidrug-resistant organisms [1]. Contact precautions include using single-patient rooms when possible, using dedicated equipment, wearing personal protective equipment (PPE), and using hand hygiene for all interactions with patients [1]. Gown use decreases the contamination of healthcare workers’ uniforms, and glove use decreases hand contamination [2]. Soap and water were found to be superior to alcohol-based hand rubs in removing *C. difficile* spores [3]. However, how to effectively teach, evaluate, and monitor proficiency in hand hygiene and PPE techniques among health personnel in hospitals remains a challenge [4].

An objective structured clinical examination (OSCE) provides a standardized setting in which skills can be evaluated [5]. The OSCE format has not been previously used to evaluate and monitor proficiency in hand hygiene and PPE use among medical students or interns.

### Purpose

The purpose of this study was to use OSCE stations at the University of Florida Medical School in the United States to provide medical interns a formative assessment of hand hygiene and the use of PPE techniques, which specifically include donning and removing a gown and gloves, hand-washing technique, and mindfulness of the contamination of clean areas with potential infectious agents. Also tested was whether feedback and teaching after OSCE testing improved these interns’ hand hygiene and PPE techniques. First, OSCE performance was compared between different groups of interns. Second, the same group of interns was compared at different stages of their training. Third, it was tested whether previous exposure to hand hygiene and PPE training during prior medical school education translated to better performance.

## Methods

### Ethics statement

This study was reviewed and approved by the University of Florida Institutional Review Board (IRB approval no., 201900575) with exempt status. According to the IRB, informed consent is not required for activities such as this, which are part of an established educational activity that will not adversely impact the learner.

### Study design

This study included not only a descriptive analysis of survey results, but also a comparative study of 2 independent groups and pretest and posttest analyses of the same group.

### Setting/participants

In the 2015–16 academic year, 123 first-year interns of the University of Florida Hospital across multiple specialties (cohort 1) participated in an OSCE in which hand hygiene and PPE was 1 of the 10 stations from February to April 2016. Cohort 1 did not receive any formal training during the academic year with regard to hand hygiene and PPE except for possible informal instruction on the wards by fellow residents or attendings. This station evaluated the ability to properly put on and take off a gown and gloves and wash hands in a scenario requiring enhanced contact precautions. A 15-item checklist was developed by our institution’s infection control team based on the Centers for Disease Control and Prevention (CDC) guidelines in conjunction with our OSCE medical educators and an infectious disease content expert [6] (Table 1). Interns also completed a brief survey about prior training in enhanced contact precautions procedures. The station used a simulated hospital room, including a sink and handwashing station, and a hallway viewed by 5 cameras from various angles. The interns read the station instructions posted on the hospital room door, which stated that the hospitalized patient had *C. difficile* colitis. The institution’s actual enhanced contact precautions door sign was also affixed to the door. Interns were video-recorded and graded based on the checklist items included in Table 1. The grader, who was trained in the proper technique using the grading rubric, observed behavior outside the room, including sanitizing hands prior to putting on gloves and technique for donning the gown. The intern then entered the simulated hospital room, followed by the grader. The intern was then prompted to take off the gown and gloves. They were evaluated on their technique for removing and disposing of the gown and gloves, as well as the effectiveness with which they washed their hands before leaving the room. Interns who did not appropriately perform any of the items were recorded as having failed the station.

Since the performance of the 123 cohort 1 interns in the pilot study was poor, this hand hygiene and PPE OSCE station was then included among the stations for the next year’s incoming interns (academic year 2016–17; cohort 2, N=151). The OSCE was administered during orientation (OSCE 1) using the same protocol as described above for the cohort 1 pilot study in July 2016. The cohort 2 interns were also asked whether they had prior training in enhanced contact precaution methods, and if so, the method by which they were trained.

### Follow-up after the interventional program

**Fig. 1.**
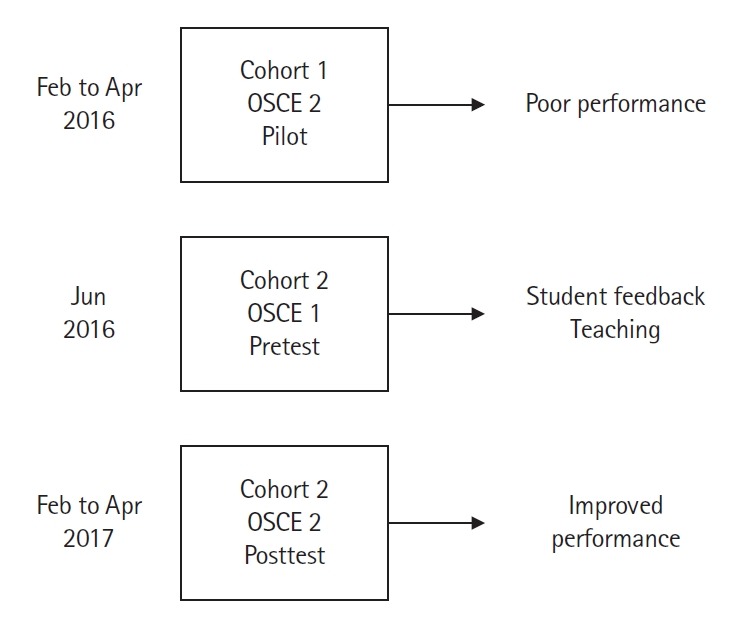
Diagram of the study including subjects, groups, and the intervention process to evaluate and monitor improvements in hand hygiene and the use of personal protective equipment techniques for interns from 2015 to 2017 at the University of Florida Health Shands Hospital, United States. OSCE, objective structured clinical examination.

In addition to assessing the cohort 2 interns’ hand hygiene and PPE skills, we introduced a 2-stage intervention to teach correct technique. First, at the end of the half-day OSCE 1 for interns, all 151 interns received their results on the gowning and gloving checklist. The interns were then shown a video created by our infection control team detailing proper technique. All interns rehearsed proper gowning and gloving procedures with immediate feedback from the infection control staff. In the 2 months following testing, the OSCE director met with each residency program to distribute individual and overall results from the OSCE to the interns and their program directors. During these meetings, she spent time reinforcing proper technique and common errors. Approximately 8 months later, in February to April 2017, during OCSE 2 for interns, cohort 2 was retested using the same checklist and standards. In total, 144 residents from cohort 2 had scores for both assessments, as 7 interns did not participate in OSCE 2 due to scheduling conflicts (Fig. 1).

### Statistical methods

The checklist items were developed using the guidelines and sequence for putting on and removing PPE published by the CDC [7]. This document was reviewed and the 15-item checklist used in this study was developed by the Intern OSCE Committee, composed of clinicians and medical educators, in consultation with infectious disease experts at the University of Florida Health Science Center/Shands Teaching Hospital. This process provided evidence of content validity for score-based inferences. The checklist items were dichotomous, scored as 1=done and 0=not done. Item scores were summed and reported as a percent correct score for the case. The internal consistency of the 3 administrations of this scenario was measured using Cronbach’s α coefficient, which is a measure of how closely related a set of items are as a group. It is considered to be a measure of scale reliability and provides further evidence of validity. Cronbach’s α was calculated for each of the 3 administrations of this assessment: for the responses of interns for OSCE 2, administered in spring 2016 (n=125), α=0.34. For OSCE 1 in summer 2016, α=0.52, and for OSCE 2 in spring 2017, α=0.34 (n=144 for both). The reliability was impacted by the high proportion of items for which all participants received credit.

Internal consistency information has been provided for completeness and so that the reliability of score differences based on the summated score from the checklists can be evaluated. However, since missing any of the checklist items would result in contamination, all were deemed critical. Therefore, a score of 100% correct was necessary to pass the station. If an intern missed any item, he or she failed the station.

The performance of the 2 cohorts was compared for each checklist item administered during the OSCE using the Mann-Whitney U-test. The difference between performance at the beginning of the intern year (OSCE 1) and 8 months later (OSCE 2) for cohort 2 was examined using the McNemar test for matched pairs and a dichotomous response (done or not done) with exact P-values. This test evaluated the null hypothesis of marginal homogeneity (i.e., whether the proportion of test takers receiving credit for an item on OSCE 1 was the same as the proportion receiving credit for that item on OSCE 2).

In addition, the performance results of those who reported prior training versus those who did not were compared using the Mann-Whitney U-test. All tests were 2-tailed, with the significance level set to α=0.05. Data analyses were conducted using IBM SPSS ver. 22.0 (IBM Corp., Armonk, NY, USA).

## Results

### Comparison of objective structured clinical examination performance after the personal protective equipment program intervention

The impact of the educational intervention was tested by comparing the performance of cohort 2 on the hand hygiene and PPE station in OSCE 1 (pretest) versus OSCE 2 (posttest). Although 151 interns participated in OSCE 1, the data analysis was limited to 144 residents for whom scores on both assessments were available. Table 1 shows the percentage of examinees who correctly performed each checklist item on the pretest (OSCE 1) and the posttest (OSCE 2), the number of examinees who passed the pretest and failed the posttest, the number who failed the pretest and passed the posttest, the P-values obtained using the McNemar test for matched pairs, the average percent correct score, and the percent passing for each testing session. The interns demonstrated significantly better performance on OSCE 2 for 8 of the 15 checklist items. The exceptions were item 1 (the intern sanitized hands prior to putting on gloves), item 5 (the intern tied gown at waist) and item 8 (the intern did not leave any items hanging exposed over the gown). The other items for which statistically significant differences were not observed (items 2, 3, 9, and 13) were those for which the proportion of successful performance in both groups was very high in the pretest, either 0.99 or 1.00, leaving no room for improvement. The mean percent correct score was significantly higher on OSCE 2 than on OSCE 1 (92% versus 77%, P<0.0001). The passing rate (perfect score) was also significantly higher (55% versus 16%). Overall, 16 interns (11.1%) passed both assessments and 58 interns (40.2%) failed both administrations. The exact Wilcoxon signed-rank test determined that there was a statistically significant difference in the proportion of interns who failed OSCE 1 and passed OSCE 2 (n=63, 43.8%) compared to the proportion who passed initially, but subsequently failed (n=7, 4.9%; W=44.8; P<0.0001) (Dataset 1).

### Difference in objective structured clinical examination performance between interns of different years

While improvements were observed from pretest to posttest for cohort 2, this could be attributed to normal and expected skill improvement through clinical experience in the hospital setting during intern year. To further evaluate whether this was the source of improvement, we next compared the end-of-year performance of the interns (cohort 1 versus cohort 2) on OSCE 2. Both cohorts were at the same stage of training and were assumed to have had similar clinical experience with enhanced contact precautions. Cohort 2 was the intervention group who had been administered the same station on OSCE 1 followed by additional training as described above. Cohort 1 served as a control group.

As shown in Table 2, the proportion of successful performance in cohort 2 was the same or higher than in cohort 1 for all items, except for item 6. Significant differences were noted for items 3, 4, 5, 7, 10, 11, 14, and 15, while 100% of the examinees in both cohorts received credit for performing items 2 and 9 correctly. Although the performance of cohort 2 was better, there remained room for improvement with respect to appropriate hand-washing (items 14 and 15): washing hands with soap and water (86% versus 95%, P=0.007) and washing hands with soap and water for at least 15 seconds (59% versus 75%, P=0.006). Even in cohort 2, 25% of examinees failed the station simply because they failed to effectively wash their hands. The mean percent correct score was significantly higher for cohort 2 than for cohort 1 (95% versus 90%, P<0.0001). More importantly, 55% of the interns in cohort 2 passed, compared to 24% in cohort 1 (P<0.0001) (Dataset 1).

### No significant effect of previous training

We also examined the impact of prior training on performance. A large proportion of interns self-reported prior training on gowning techniques as part of enhanced contact precautions (cohort 1: 50 [42.0%]; cohort 2: 97 [67.4%]). For cohort 2, we added the method of prior training to the survey, and 106 interns (73.6%) reported that they had observed others on the wards, 17 (11.8%) had either a one-on-one demonstration or formal teaching session, 6 (4.2%) had watched a video presentation, and 15 (10.4%) listed an “other” method of learning. We found that interns who reported previous training did not perform significantly better on the OSCE station (Dataset 2 and Dataset 3).

## Discussion

### Key results

Our study showed that an OSCE could be utilized as a formative assessment tool in order to provide directed, hands-on training in hand hygiene and PPE to prevent *C. difficile* transmission. Multiple evaluation steps were included based on errors noted in the literature and seen in practice to serve as a training checklist so that the proper technique is learned. This allowed the interns not only to reflect on methodology, but also to be mindful of contamination sources. The fact that OSCEs are observed is an inherent advantage in terms of their ability to demonstrate the gaps in knowledge and technical deficiencies, allowing for more targeted teaching. In this situation, an OSCE was used to quantitatively monitor skill improvement by demonstrating a statistically significant positive change in performance, even 8 months after training took place. We are not aware of any previous study that utilized an OSCE to both evaluate and monitor hand hygiene and PPE skills.

### Comparison with previous relevant studies

Previous studies have shown that healthcare workers often employ incorrect practices of hand hygiene and PPE use [7]. Several techniques have been used to evaluate compliance with varying success, such as checking *C. difficile* spore counts on hands [8], using covert observers (“secret shoppers”) [9], and simulations with florescent lotion [10]. A survey of healthcare workers showed that the most common method of instruction was on-the-job training by coworkers or supervisors. Overall, demonstrating competence in enhanced contact precautions is not required [11].

### Interpretation and suggestions

Our protocol incorporated an immediate feedback method followed by video instruction on the correct technique, and then practice using that technique in the same OSCE setting. The instruction was reinforced within the following 2 months using additional didactics. Although there was no direct control group that did not receive immediate feedback and training, the pilot study (cohort 1) did demonstrate that residents without training performed poorly in hand hygiene and PPE in their OSCE at the end of their first year. It appears that an empirical watch-and-learn methodology with extended exposure during inpatient rotations does not result in improved proficiency.

John et al. [10] reported that only 7% of the 22 medical students they tested exhibited the correct gowning and gloving technique. Our study confirmed this finding with a larger sample size, demonstrating that most students were not adequately trained in enhanced contact precaution during medical school. We found no significant correlation between interns having received enhanced contact precaution training in medical school and their proficiency upon entering residency. Since the residents came from different medical schools throughout the nation, this suggests that the problem is likely not isolated. Given how many hours third- and fourth-year medical students are involved in direct patient care, their inadequate proficiency in contact precautions may be a factor contributing to the increasing frequency of healthcare-associated *C. difficile* infections. Our study also showed that persistent errors occurred in hand-washing despite OSCE training. Even with proper PPE use, without proper hand-washing afterwards, *C. difficile* transmission can still occur.

Future studies are needed with an increased emphasis on the hand-washing portion of the teaching workshop. Adding florescent lotion to the gloves during the OSCE may also increase assessment accuracy [10]. A follow-up study to evaluate whether improvements in technique shown through an OSCE translate to daily patient care interactions and, ultimately, whether it affects the transmission of *C. difficile* or multidrug-resistant organisms would be a helpful next step. If effective, this type of training could certainly be expanded to medical students, possibly by including it as part of their step 2 clinical skills examination and during orientation, and could also be included in continuing education for clinicians.

### Limitation

The fact that this study was centered on a single OSCE station certainly limits its reliability. The study was also not designed to demonstrate a direct correlation between OSCE training and institutional reduction of the *C. difficile* infection rate.

### Generalizability

Although this was a single-institute study, it may be adopted by other medical schools in the United States because the OSCE items used in this study were modified from the CDC guideline on the proper sequence of putting on and taking off PPE [6].

### Conclusion

In this study, the effectiveness of using an OSCE to evaluate and monitor hand hygiene and PPE proficiency among interns in the United States was tested. It was found that OSCE is a valuable method for this purpose. It is recommended that OSCEs be adopted to improve the performance of interns or trainees in hospitals.

## Figures and Tables

**Table 1.** *Clostridioides difficile* hand hygiene and personal protective equipment use for cohort 2: paired items of intern OSCE 1 versus intern OSCE 2 (n=144)

Item	% Correct		Frequency		P-value: McNemar test for matched pairs
	Pretest	Posttest	Negative differences (a)	Positive differences (b)	
1. The intern sanitized hands prior to putting on gloves.	0.903	0.965	5	14	0.039^*^
2. The intern donned the gown.	1.000	1.000	0	0	1.000
3. The intern put his/her head through the gown opening.	0.986	1.000	0	2	0.157
4. The intern put his/her thumbs through the loops	0.826	0.993	1	25	0.000^*^
5. The intern tied the gown at the waist. (This can be done later but must be done before walks in the room)	0.972	1.000	0	4	0.046^*^
6. The intern donned gloves after gowning.	0.889	0.951	3	12	0.020^*^
7. The intern pulled the gloves up over the cuffs of the gown.	0.708	0.924	4	35	0.000^*^
8. The intern did not leave any items hanging exposed over the gown.	0.868	0.938	9	19	0.059
9. The intern removed the gown and gloves while in the patient room.	1.000	1.000	0	0	1.000
10. The intern removed gown by pulling from the front. Their hands must stay in the front even if [they] don’t have gloves on.	0.903	0.965	4	13	0.029^*^
11. The intern folded or rolled the gown into a bundle while taking it off.	0.507	0.924	4	64	0.000^*^
12. The intern removed gloves while removing gown.	0.792	0.951	4	27	0.000^*^
13. The intern discarded gown and gloves in garbage can inside room.	0.993	1.000	0	1	0.317
14. The intern washed hands with soap and water.	0.868	0.951	4	16	0.007^*^
15. The intern washed hands with soap and water for at least 15 seconds.	0.486	0.75	13	51	0.000^*^
Case average percent	84.676	95.4167			0.000 (Z=-8.168)
Case pass (100% required)	16.0% (n=23)	54.9% (n=79)			0.000 (Z=-6.693)

(a): number of interns who passed the pretest item, but failed the posttest item; (b): number of interns who failed the pretest item, but passed the post-test item. OSCE, objective structured clinical examination.

* P<0.05; items where the difference in percent correct is significant.

**Table 2.** *Clostridioides difficile* hand hygiene and personal protective equipment use: a comparison of intern OSCE 1 between cohorts 1 and 2

Item	% Correct		P-value^a)^
	Cohort 1 (n=125; on-the-job training)	Cohort 2 (n=144; training after OSCE 1)	
1. The intern sanitized hands prior to putting on gloves.	0.97	0.97	0.902
2. The intern donned the gown.	1.00	1.00	1.000
3. The intern put his/her head through the gown opening.	0.97	1.00	0.031^*^
4. The intern put his/her thumbs through the loops.	0.95	0.99	0.035^*^
5. The intern tied the gown at the waist. (This can be done later but must be done before walks in the room)	0.96	1.00	0.016^*^
6. The intern donned gloves after gowning.	0.97	0.95	0.493
7. The intern pulled the gloves up over the cuffs of the gown.	0.81	0.92	0.005^*^
8. The intern did not leave any items hanging exposed over the gown.	0.94	0.94	0.960
9. The intern removed the gown and gloves while in the patient room.	1.00	1.00	1.000
10. The intern removed gown by pulling from the front. Their hands must stay in the front even if [they] don’t have gloves on.	0.78	0.97	0.000^*^
11. The intern folded or rolled the gown into a bundle while taking it off.	0.79	0.92	0.002^*^
12. The intern removed gloves while removing gown.	0.90	0.95	0.085
13. The intern discarded gown and gloves in garbage can inside room.	0.98	1.00	0.128
14. The intern washed hands with soap and water.	0.86	0.95	0.007^*^

OSCE, objective structured clinical examination.

* P≤0.05; items where the difference in percent correct is significant.

a) P-values are for the results of significance testing at 0.05 using the 2-tailed Mann-Whitney U-test.

## Footnotes

**Data availability** Data files are available from https://doi.org/10.7910/DVN/6SVFUJ Dataset 1. Interns’ proficiency of hand hygiene and personal protective equipment checklist score Dataset 2. Cohort2 OSCE 1 survey data Dataset 3. Cohort1 OSCE 2 survey data

## Supplementary materials

Data files are available from https://doi.org/10.7910/DVN/6SVFUJ

Supplement 1. Audio recording of the abstract.
